# Adaptive Predefined-Time Tracking Control for Robotic Manipulator Based on Actor-Critic Reinforcement Learning

**DOI:** 10.3390/s26051529

**Published:** 2026-02-28

**Authors:** Yong Qin, Yuan Sun, Jun Huang, Yankai Li

**Affiliations:** 1 School of Artificial Intelligence and Smart Manufacturing, Hechi University, Hechi 546300, China; 05005@hcnu.edu.cn; 2 School of Mechanical and Electrical Engineering, Soochow University, Suzhou 215131, China; cauchyhot@163.com; 3 School of Automation and Information Engineering, Xi’an University of Technology, Xi’an 710048, China

**Keywords:** predefined-time control, actor-critic reinforcement learning, adaptive neural network control, backstepping control

## Abstract

This paper proposes a novel predefined-time adaptive neural tracking control method for uncertain manipulator systems based on Actor-Critic reinforcement learning framework. The proposed control scheme integrates the advantages of predefined-time stability theory and reinforcement learning to achieve fast convergence with guaranteed settling time bounds while handling unknown system dynamics. An Actor neural network is designed to approximate the unknown nonlinear functions and generate control inputs, while a Critic neural network evaluates the cost-to-go function to guide the learning process. The predefined-time convergence is ensured by incorporating specially designed terms into both the control law and the neural network weight update laws. The upper bound of the settling time can be explicitly preset by a single design parameter, independent of initial conditions and system parameters. Rigorous stability analysis based on Lyapunov theory proves that all closed-loop signals are bounded and the tracking error converges to a small neighborhood of the origin within the predefined time. Simulation results on a single-link manipulator system demonstrate the effectiveness and superiority of the proposed control scheme compared with conventional PID control.

## 1. Introduction

Robotic manipulators have been extensively deployed in industrial manufacturing, medical surgery, space exploration, and military applications due to their high flexibility, precision, repeatability, and efficiency [1,2]. In these applications, the control system must achieve accurate trajectory tracking while adapting to varying operating conditions and task requirements. However, the control of robotic manipulators remains challenging due to their inherent nonlinearities arising from trigonometric functions in the dynamic equations, strong coupling effects between joints, and inevitable uncertainties stemming from unmodeled dynamics, parameter variations, friction, and external disturbances [3]. Therefore, developing advanced control strategies that simultaneously guarantee tracking performance, fast convergence, and robustness against uncertainties has become a critical research topic in the field of robotics and control engineering.

To address the challenges posed by system uncertainties, numerous advanced control strategies have been developed over the past decades. Adaptive control provides an effective approach to handle parametric uncertainties through online parameter estimation, enabling the controller to adjust its parameters in real-time based on system behavior [4,5]. Neural network (NN)-based control has gained significant attention for its universal approximation capability, which allows it to deal with unknown nonlinear functions without requiring explicit mathematical models [6]. The combination of adaptive control and neural networks, known as adaptive neural network control, has demonstrated excellent performance in handling both parametric and functional uncertainties, and has been successfully applied to various robotic systems [7,8]. Despite these advances, most existing adaptive neural control methods only guarantee asymptotic or exponential convergence, where the settling time depends on initial conditions and system parameters, which may not satisfy the strict timing requirements in practical applications.

In practical robotic applications, fast convergence is often a critical requirement, particularly in time-critical tasks such as assembly operations, surgical procedures, and emergency response scenarios. To achieve convergence in finite time, finite-time control and fixed-time control have been developed based on nonsmooth Lyapunov analysis [9,10]. Finite-time control ensures that the system states converge to the equilibrium within a finite settling time, but this settling time depends on initial conditions, making it difficult to predict or prescribe in advance. Fixed-time control addresses this limitation by ensuring that the settling time is bounded regardless of initial conditions [3]. However, the relationship between the settling time bound and control parameters in fixed-time control is implicit and complex, typically involving multiple design parameters in a nonlinear manner, which complicates the controller tuning process for achieving desired convergence speed.

Recently, predefined-time control has emerged as a promising approach that allows designers to explicitly preset the upper bound of the settling time through a single design parameter [11,12,13]. This feature is particularly attractive for applications with strict timing requirements, as the maximum convergence time can be directly specified according to task demands without complex parameter calculations. Several predefined-time control schemes have been proposed for various systems including rigid spacecraft attitude stabilization [14] and robotic manipulators [15]. However, most existing predefined-time control methods require accurate system models or assume that the system uncertainties are bounded with known bounds, which significantly limits their practical applicability to real-world robotic systems where model parameters are often unknown or time-varying.

On the other hand, reinforcement learning (RL) has shown great potential in control applications due to its ability to learn optimal control policies through interaction with the environment without requiring explicit system models [16,17]. Among various RL architectures, the Actor-Critic (AC) framework is particularly well-suited for continuous control problems, where the Actor network generates control actions and the Critic network evaluates the performance by estimating the value function or cost-to-go [18,19]. The combination of Actor-Critic reinforcement learning and neural network approximation has been successfully applied to various robotic control problems, demonstrating improved adaptability and optimality compared to conventional methods [20,21]. The Actor-Critic structure offers several advantages: the Critic provides a global performance metric for guiding the Actor’s learning, the dual-network architecture separates policy evaluation from policy improvement for enhanced learning efficiency, and the framework naturally accommodates online learning in real-time control scenarios.

Despite the significant progress in each individual area, there remains a gap in the literature regarding the unified treatment of predefined-time convergence, adaptive learning capability, and optimal control for uncertain robotic systems. Most existing predefined-time control methods lack the ability to handle unknown nonlinearities adaptively, while conventional adaptive neural control schemes cannot guarantee predefined-time convergence. The integration of predefined-time stability with Actor-Critic reinforcement learning presents unique theoretical challenges: the predefined-time convergence mechanism must be incorporated into both the control law and the neural network weight update laws in a compatible manner, and the stability analysis must account for the coupled dynamics of tracking errors and weight estimation errors within the predefined-time framework. To the best of the authors’ knowledge, the problem of predefined-time adaptive neural control using Actor-Critic reinforcement learning for robotic manipulators has not been adequately addressed in the existing literature.

Motivated by the above observations, this paper proposes a novel predefined-time adaptive neural tracking control scheme for uncertain single-link manipulator systems based on the Actor-Critic reinforcement learning framework. The main contributions of this paper are summarized as follows:

- A novel control framework that synergistically integrates predefined-time stability theory with Actor-Critic reinforcement learning is proposed. The Actor neural network approximates unknown system dynamics and generates control inputs, while the Critic neural network evaluates the cost-to-go function to guide the learning process, achieving both guaranteed convergence time and online learning capability.
- Predefined-time neural network weight update laws are designed with specially constructed terms that incorporate the predefined-time convergence mechanism. These update laws ensure the convergence of both tracking errors and weight estimation errors within the predefined time while maintaining the learning and approximation capabilities of the neural networks.
- The upper bound of the settling time can be explicitly preset by a single design parameter $T_{c}$, satisfying $T_{P}<\sqrt{2}T_{c}$, which is independent of initial conditions and system parameters. This explicit relationship between the design parameter and settling time bound greatly simplifies the controller design process for applications with specific timing requirements.

The remainder of this paper is organized as follows. Section 2 presents the single-link manipulator system model, introduces the Actor-Critic neural network framework, and provides necessary mathematical preliminaries including the predefined-time stability lemma. Section 3 details the controller design procedure, including the predefined-time virtual controller, the Actor-Critic reinforcement learning controller, and the predefined-time weight update laws. Section 4 provides the rigorous stability analysis based on Lyapunov theory. Section 5 presents comprehensive simulation results to validate the effectiveness and superiority of the proposed control scheme. Finally, Section 6 concludes the paper and discusses future research directions.

## 2. Preliminaries and Problem Formulation

### 2.1. System Model

Consider a single-link robotic manipulator system described by the following dynamic equation:

(1) $$I\ddot{q}+c_{0}\dot{q}+mgl\cos\left(q\right)=\tau +d$$

where q∈R denotes the joint angle, $\dot{q}\in R$ is the angular velocity, $\ddot{q}\in R$ is the angular acceleration, τ∈R represents the control torque, $I=\frac{4}{3}ml^{2}$ is the moment of inertia, *m* is the link mass, *l* is the link length, $c_{0}$ is the viscous friction coefficient, *g* is the gravitational acceleration, and *d* represents the bounded external disturbance satisfying $\left|d\right|\leq \bar{d}$ with $\bar{d}$ being a known positive constant.

Define the state variables $x_{1}=q$ and $x_{2}=\dot{q}$. The system (1) can be rewritten in the following state-space form:

(2) $$\left\{\begin{array}{c} {\dot{x}}_{1}=x_{2}\\ {\dot{x}}_{2}=f\left(x\right)+g_{1}u\\ y=x_{1} \end{array}\right.$$

where u=τ is the control input, $y=x_{1}$ is the system output, $g_{1}=\frac{1}{I}$ is a known positive constant, and

(3) $$f\left(x\right)=-\frac{1}{I}\left[c_{0}x_{2}+mgl\cos\left(x_{1}\right)-d\right]$$

is an uncertain nonlinear function.

**Assumption** **1.** *The desired reference trajectory $y_{d}$ and its derivatives ${\dot{y}}_{d}$, ${\ddot{y}}_{d}$ are continuous and bounded, i.e., there exist positive constants ${\bar{y}}_{d}$, ${\bar{y}}_{d1}$, ${\bar{y}}_{d2}$ such that $|y_{d}|\leq {\bar{y}}_{d}$, $|{\dot{y}}_{d}|\leq {\bar{y}}_{d1}$, $|{\ddot{y}}_{d}|\leq {\bar{y}}_{d2}$.*

### 2.2. Control Objective

The control objective is to design an adaptive neural tracking controller based on Actor-Critic reinforcement learning such that:

(i) The joint angle $x_{1}$ tracks the desired trajectory $y_{d}$ with the tracking error converging to a small neighborhood of the origin within a predefined time $T_{P}<\sqrt{2}T_{c}$, where $T_{c}$ is a preset design parameter.
(ii) All signals in the closed-loop system remain bounded within the predefined time.
(iii) The Actor-Critic neural networks learn to compensate for the unknown system dynamics online.

### 2.3. Actor-Critic Neural Network Framework

To handle the unknown nonlinear functions in the system and achieve adaptive optimal control, this paper employs an Actor-Critic reinforcement learning framework. This framework consists of two cooperatively working neural networks: the Actor network is responsible for approximating unknown dynamics and generating control policies, while the Critic network evaluates the control performance and guides the Actor’s learning process.

#### 2.3.1. RBF Basis Function

Both neural networks adopt Radial Basis Functions (RBFs) as basis functions due to their universal approximation capability. For a continuous function $h\left(Z\right):R^{n}\to R$ defined on a compact set $\Omega_{Z}\subset R^{n}$, it can be approximated by an RBF neural network as:

(4) $$h\left(Z\right)=W^{*T}S\left(Z\right)+\epsilon \left(Z\right)$$

where $Z={\left[Z_{1},Z_{2},\ldots ,Z_{n}\right]}^{T}\in \Omega_{Z}$ is the input vector, $W^{*}={\left[w_{1}^{*},w_{2}^{*},\ldots ,w_{l}^{*}\right]}^{T}\in R^{l}$ is the ideal weight vector, *l* is the number of neural network nodes, $S\left(Z\right)={\left[s_{1}\left(Z\right),s_{2}\left(Z\right),\ldots ,s_{l}\left(Z\right)\right]}^{T}$ is the basis function vector, and ϵ(Z) is the approximation error satisfying $\left|\epsilon \left(Z\right)\right|\leq \bar{\epsilon }$.

The Gaussian function is employed as the basis function:

(5) $$s_{i}\left(Z\right)=\exp\left(-\frac{∥Z-\mu_{i}{∥}^{2}}{\eta^{2}}\right),\;i=1,2,\ldots ,l$$

where $\mu_{i}={\left[\mu_{i1},\mu_{i2},\ldots ,\mu_{in}\right]}^{T}$ is the center of the *i*-th basis function, and η>0 is the width of the Gaussian function.

#### 2.3.2. Critic Network Structure

The Critic network is designed to evaluate the long-term performance of the current control policy. The long-term cost function is defined as:

(6) $$I\left(t\right)=\int_{t}^{\infty }e^{-\frac{m-t}{\psi }}\phi \left(m\right)\;dm$$

where ψ>0 is the discount factor, and the instantaneous cost function is defined as:

(7) $$\phi \left(t\right)=z_{1}^{T}Dz_{1}+\tau^{T}R\tau$$

where D>0 and R>0 are positive definite weight matrices that penalize the tracking error and control effort, respectively.

Using the RBF neural network to approximate the cost function:

(8) $$I=W_{c}^{*T}S_{c}\left(Z_{c}\right)+\epsilon_{c}$$

(9) $$\hat{I}={\hat{W}}_{c}^{T}S_{c}\left(Z_{c}\right)$$

where $Z_{c}=z_{1}$ is the Critic network input, $W_{c}^{*}\in R^{l_{c}}$ is the ideal weight vector, ${\hat{W}}_{c}$ is the estimated weight vector, $S_{c}\left(Z_{c}\right)$ is the basis function vector, and $\epsilon_{c}$ satisfies $|\epsilon_{c}|\leq {\bar{\epsilon }}_{c}$.

When ψ→∞, based on the Bellman equation, the temporal difference (TD) error can be expressed as:

(10) $$\gamma \left(t\right)=\phi \left(t\right)+\dot{\hat{I}}\left(t\right)=\phi \left(t\right)+{\hat{W}}_{c}^{T}\Lambda$$

where $\Lambda =-\frac{S_{c}}{\psi }+\nabla S_{c}{\dot{Z}}_{c}$. The learning objective of the Critic network is to minimize the TD error.

#### 2.3.3. Actor Network Structure

The Actor network is designed to approximate the unknown nonlinear functions in the system and assist in generating control inputs. Define the composite unknown function:

(11) $$\varphi =f\left(x\right)-{\dot{\alpha }}_{1}+z_{1}+\frac{1}{2}z_{2}$$

where f(x) is the unknown nonlinear term of the system, and ${\dot{\alpha }}_{1}$ is the derivative of the virtual control.

Using the RBF neural network to approximate φ:

(12) $$\varphi =W_{a}^{*T}S_{a}\left(Z_{a}\right)+\epsilon_{a}$$

(13) $$\hat{\varphi }={\hat{W}}_{a}^{T}S_{a}\left(Z_{a}\right)$$

where $Z_{a}={\left[x_{1},x_{2},y_{d},{\dot{y}}_{d},{\ddot{y}}_{d}\right]}^{T}$ is the Actor network input, $W_{a}^{*}\in R^{l_{a}}$ is the ideal weight vector, ${\hat{W}}_{a}$ is the estimated weight vector, $S_{a}\left(Z_{a}\right)$ is the basis function vector, and $\epsilon_{a}$ satisfies $|\epsilon_{a}|\leq {\bar{\epsilon }}_{a}$.

#### 2.3.4. Actor-Critic Cooperative Learning Mechanism

The cooperative learning mechanism of the Actor-Critic framework operates as follows:

(1) Critic evaluates policy performance: The Critic network computes the estimated cost function $\hat{I}$ based on the current state and control input, evaluating the quality of the Actor’s current policy. A larger $\hat{I}$ indicates poorer policy performance that requires improvement.
(2) Actor improves control policy: The Actor network utilizes the evaluation information $\hat{I}$ provided by the Critic as feedback to adjust its weights ${\hat{W}}_{a}$, thereby improving the control policy to minimize the long-term cost.
(3) Online cooperative update: The weights of both networks are updated in real-time during the control process. Through continuous “evaluation-improvement” cycles, the control performance is progressively optimized.

Define the weight estimation errors as:

(14) $${\tilde{W}}_{c}=W_{c}^{*}-{\hat{W}}_{c},\;{\tilde{W}}_{a}=W_{a}^{*}-{\hat{W}}_{a}$$

The specific weight update laws for the Actor-Critic networks will be designed in Section 3, incorporating the predefined-time stability requirements.

**Remark** **1.** *Compared with traditional single neural network adaptive control, the Actor-Critic framework possesses the following advantages: (i) The value function evaluation provided by the Critic offers a global performance metric for the Actor, rather than relying solely on local error information; (ii) The dual-network structure separates policy evaluation from policy improvement, enhancing learning efficiency and stability; (iii) This framework is naturally suited for integration with predefined-time control, allowing the predefined-time convergence mechanism to be incorporated into the weight update laws of both networks.*

### 2.4. Technical Lemmas

**Lemma** **1** ([22])**.** *For any ξ∈R and ρ>0, the following inequality holds:*

(15) $$0\leq \left|\xi \right|-\xi \tanh\left(\frac{\xi }{\rho }\right)\leq \delta \rho$$

*where δ=0.2785.*

**Lemma** **2** ([23])**.** *For $x_{i}\geq 0$ (i=1,2,…,n) and γ>0, the following inequalities hold:*

(16) $$\sum_{i=1}^{n}x_{i}^{\gamma }\geq {\left(\sum_{i=1}^{n}x_{i}\right)}^{\gamma },\;0<\gamma <1$$

(17) $$\sum_{i=1}^{n}x_{i}^{\gamma }\geq n^{1-\gamma }{\left(\sum_{i=1}^{n}x_{i}\right)}^{\gamma },\;\gamma >1$$

**Lemma** **3.** *For y≥x and v>1, the following inequality holds:*

(18) $$x{\left(y-x\right)}^{v}\leq \frac{v}{1+v}\left(y^{1+v}-x^{1+v}\right)$$

**Lemma** **4** ([24])**.** *(Predefined-Time Stability) Consider the system $\dot{x}=f\left(t,x\right)$. If there exists a continuous positive definite function V(x) and parameters 0<β<1, $T_{c}>0$, 0<σ<∞ such that*

(19) $$\dot{V}\left(x\right)\leq -\frac{\pi }{\beta T_{c}}\left(V^{1-\frac{\beta }{2}}+V^{1+\frac{\beta }{2}}\right)+\sigma$$

*then the system is practically predefined-time stable (PPTS), and the convergence region is*

(20) $$\left\{\lim_{t\to T_{P}}x|V\leq \min\left\{{\left(\frac{2\beta T_{c}\sigma }{\pi }\right)}^{\frac{2}{2-\beta }},{\left(\frac{2\beta T_{c}\sigma }{\pi }\right)}^{\frac{2}{2+\beta }}\right\}\right\}$$

*where $T_{P}$ is the settling time satisfying $T_{P}<T_{\max}=\sqrt{2}T_{c}$.*

**Remark** **2.** *Lemma 4 is fundamental to predefined-time stability theory. The key feature is that the upper bound of the settling time $T_{\max}=\sqrt{2}T_{c}$ can be explicitly preset through the parameter $T_{c}$, independent of the initial conditions and system parameters. This is in contrast to finite-time control where the settling time depends on initial conditions, and fixed-time control where the settling time bound is implicitly determined by multiple parameters.*

**Lemma** **5** ([25])**.** *For any z∈R and κ>0:*

(21) $$0\leq \left|z\right|-\frac{z^{2}}{\sqrt{z^{2}+\kappa^{2}}}<\kappa$$

**Lemma** **6.** *(Power Function Inequality) For any x>0 and 0<β<1, the following inequality holds:*

(22) $$x^{2}\geq x^{2-\beta }-C_{\beta }$$

*where $C_{\beta }=\frac{\beta }{2}{\left(\frac{2-\beta }{2}\right)}^{\frac{2-\beta }{\beta }}$ is a positive constant depending only on β.*

**Proof.**  Define $f\left(x\right)=x^{2}-x^{2-\beta }$ for x>0. Taking the derivative:

$$f\prime \left(x\right)=2x-\left(2-\beta \right)x^{1-\beta }=x^{1-\beta }\left(2x^{\beta }-\left(2-\beta \right)\right)$$

Setting f′(x)=0 yields the critical point $x^{*}={\left(\frac{2-\beta }{2}\right)}^{\frac{1}{\beta }}$. Since $f\prime \prime \left(x^{*}\right)>0$, this is a minimum point. The minimum value is:

$$f\left(x^{*}\right)=-\frac{\beta }{2}{\left(\frac{2-\beta }{2}\right)}^{\frac{2-\beta }{\beta }}=-C_{\beta }$$

Therefore, $f\left(x\right)\geq -C_{\beta }$, which completes the proof. □

## 3. Actor-Critic Predefined-Time Controller Design

**Figure 1:**
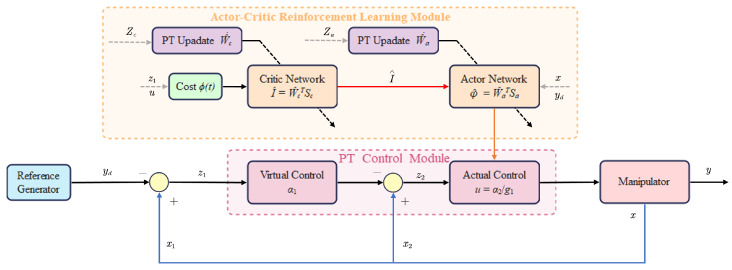
Block diagram of the Actor-Critic predefined-time control system.

In this section, we present the design of the predefined-time adaptive neural tracking controller based on the Actor-Critic reinforcement learning framework. The control system architecture is illustrated in Figure 1. The Actor network receives system states and reference signals, outputs the control signal to compensate for unknown dynamics. The Critic network evaluates the cost-to-go and provides feedback to guide the Actor’s learning process. Both networks are updated using predefined-time weight update laws.

### 3.1. Predefined-Time Virtual Controller Design

Define the tracking error variables as:

(23) $$z_{1}=x_{1}-y_{d}$$

(24) $$z_{2}=x_{2}-\alpha_{1}$$

where $\alpha_{1}$ is the virtual control law to be designed.

The time derivative of $z_{1}$ is:

(25) $${\dot{z}}_{1}={\dot{x}}_{1}-{\dot{y}}_{d}=x_{2}-{\dot{y}}_{d}=z_{2}+\alpha_{1}-{\dot{y}}_{d}$$

Design the predefined-time virtual controller as:

(26) $$\alpha_{1}=-\frac{z_{1}{\overset{ˇ}{\alpha }}_{1}^{2}}{\sqrt{z_{1}^{2}{\overset{ˇ}{\alpha }}_{1}^{2}+\varepsilon_{1}^{2}}}+{\dot{y}}_{d}$$

where $\varepsilon_{1}>0$ is a small positive constant, and

(27) $${\overset{ˇ}{\alpha }}_{1}=\frac{\pi }{\beta T_{c}}\left[2^{\beta }{\left(\frac{1}{2}\right)}^{1+\frac{\beta }{2}}{\mathrm{sig}}^{1+\beta }\left(z_{1}\right)+{\left(\frac{1}{2}\right)}^{1-\frac{\beta }{2}}{\mathrm{sig}}^{1-\beta }\left(z_{1}\right)\right]$$

with 0<β<1, $T_{c}>0$ being the predefined time parameter, and ${\mathrm{sig}}^{\gamma }\left(a\right)={\left|a\right|}^{\gamma }\mathrm{sgn}\left(a\right)$.

**Remark** **3.** *The virtual controller (26) is specifically designed to achieve predefined-time convergence. The structure $-\frac{z_{1}{\overset{ˇ}{\alpha }}_{1}^{2}}{\sqrt{z_{1}^{2}{\overset{ˇ}{\alpha }}_{1}^{2}+\varepsilon_{1}^{2}}}$ ensures that the derivative ${\dot{\alpha }}_{1}$ remains bounded even when $z_{1}$ approaches zero, thus avoiding the singularity issue that commonly arises in traditional finite-time control designs where terms like ${\mathrm{sig}}^{1-\beta }\left(z_{1}\right)$ with 0<β<1 would cause unbounded derivatives.*

Consider the Lyapunov function candidate:

(28) $$V_{1}=\frac{1}{2}z_{1}^{2}$$

Taking the time derivative of $V_{1}$ and substituting (25) and (26):

(29) $${\dot{V}}_{1}=z_{1}{\dot{z}}_{1}=z_{1}\left(z_{2}+\alpha_{1}-{\dot{y}}_{d}\right)=z_{1}z_{2}-\frac{z_{1}^{2}{\overset{ˇ}{\alpha }}_{1}^{2}}{\sqrt{z_{1}^{2}{\overset{ˇ}{\alpha }}_{1}^{2}+\varepsilon_{1}^{2}}}$$

Applying Lemma 5:

(30) $$-\frac{z_{1}^{2}{\overset{ˇ}{\alpha }}_{1}^{2}}{\sqrt{z_{1}^{2}{\overset{ˇ}{\alpha }}_{1}^{2}+\varepsilon_{1}^{2}}}<\varepsilon_{1}-\left|z_{1}{\overset{ˇ}{\alpha }}_{1}\right|<\varepsilon_{1}-\frac{\pi }{\beta T_{c}}{\left(\frac{z_{1}^{2}}{2}\right)}^{1-\frac{\beta }{2}}-\frac{\pi }{\beta T_{c}}2^{\beta }{\left(\frac{z_{1}^{2}}{2}\right)}^{1+\frac{\beta }{2}}$$

Therefore:

(31) $${\dot{V}}_{1}<-\frac{\pi }{\beta T_{c}}{\left(\frac{z_{1}^{2}}{2}\right)}^{1-\frac{\beta }{2}}-\frac{\pi }{\beta T_{c}}2^{\beta }{\left(\frac{z_{1}^{2}}{2}\right)}^{1+\frac{\beta }{2}}+z_{1}z_{2}+\varepsilon_{1}$$

### 3.2. Actor-Critic Reinforcement Learning Controller Design

The time derivative of $z_{2}$ is:

(32) $${\dot{z}}_{2}={\dot{x}}_{2}-{\dot{\alpha }}_{1}=f\left(x\right)+g_{1}u-{\dot{\alpha }}_{1}$$

Define the unknown nonlinear function:

(33) $$\varphi =f\left(x\right)-{\dot{\alpha }}_{1}+z_{1}+\frac{1}{2}z_{2}$$

#### 3.2.1. Critic Network Design

The Critic network is designed to approximate the cost-to-go function and evaluate the control performance. Define the long-term cost function:

(34) $$I\left(t\right)=\int_{t}^{\infty }e^{-\frac{m-t}{\psi }}\phi \left(m\right)dm$$

where ψ>0 is a discount factor, and the instantaneous cost function is defined as:

(35) $$\phi \left(t\right)=z_{1}^{T}Dz_{1}+\tau^{T}R\tau$$

with D>0 and R>0 being positive definite weight matrices.

The cost-to-go function is approximated by the Critic neural network:

(36) $$I=W_{c}^{*T}S_{c}\left(Z_{c}\right)+\epsilon_{c}$$

(37) $$\hat{I}={\hat{W}}_{c}^{T}S_{c}\left(Z_{c}\right)$$

where $Z_{c}=z_{1}$ is the Critic network input, $W_{c}^{*}$ is the ideal weight vector, ${\hat{W}}_{c}$ is the estimated weight vector, $S_{c}\left(Z_{c}\right)$ is the basis function vector, and $\epsilon_{c}$ is the approximation error.

When ψ→∞, the temporal difference error can be expressed as:

(38) $$\gamma \left(t\right)=\phi \left(t\right)+\dot{\hat{I}}\left(t\right)=\phi \left(t\right)+\nabla \hat{I}\left(t\right){\dot{Z}}_{c}$$

The predefined-time Critic network weight update law is designed as:

(39) $${\dot{\hat{W}}}_{c}=-\sigma_{c}\left(\phi \left(t\right)+{\hat{W}}_{c}^{T}\Lambda \right)\Lambda -\varsigma_{c}{\hat{W}}_{c}-c_{c}{\left|{\hat{W}}_{c}\right|}^{\beta }{\hat{W}}_{c}$$

where $\Lambda =-\frac{S_{c}}{\psi }+\nabla S_{c}{\dot{Z}}_{c}$, $\sigma_{c}>0$ is the learning rate, $\varsigma_{c}=\frac{\pi \cdot 2^{\frac{\beta }{2}}\cdot r_{c}^{\frac{\beta }{2}}}{\beta T_{c}}$, $c_{c}=\frac{\pi (2+\beta )}{2\beta T_{c}\left(1+\beta \right)r_{c}^{\frac{\beta }{2}}}$ and $r_{c}>0$ is a design parameter.

#### 3.2.2. Actor Network Design

The Actor network is designed to approximate the unknown function φ and generate control inputs. Using RBFNN approximation:

(40) $$\varphi =W_{a}^{*T}S_{a}\left(Z_{a}\right)+\epsilon_{a}$$

where $Z_{a}={\left[x_{1},x_{2},y_{d},{\dot{y}}_{d},{\ddot{y}}_{d}\right]}^{T}$ is the Actor network input, $W_{a}^{*}$ is the ideal weight vector, $S_{a}\left(Z_{a}\right)$ is the basis function vector, and $|\epsilon_{a}|\leq {\bar{\epsilon }}_{a}$.

The predefined-time Actor network weight update law is designed as:

(41) $${\dot{\hat{W}}}_{a}=r_{a}z_{2}g_{1}S_{a}\tanh\left(\frac{z_{2}g_{1}∥S_{a}∥}{\rho }\right)-\varsigma_{a}{\hat{W}}_{a}-c_{a}{\left|{\hat{W}}_{a}\right|}^{\beta }{\hat{W}}_{a}+K_{I}\hat{I}S_{a}$$

where $r_{a}>0$ is the learning rate, $\varsigma_{a}=\frac{\pi \cdot 2^{\frac{\beta }{2}}\cdot r_{a}^{\frac{\beta }{2}}}{\beta T_{c}}$, $c_{a}=\frac{\pi (2+\beta )}{2\beta T_{c}\left(1+\beta \right)r_{a}^{\frac{\beta }{2}}}$, $K_{I}>0$ is the Critic feedback gain and ρ>0 is a design parameter.

**Remark** **4.** *The weight update laws (39) and (41) ensure both learning capability and predefined-time convergence by incorporating three essential terms: the first is the standard gradient descent term, which minimizes the approximation or temporal difference error; the second term, $-\varsigma \hat{W}$, introduces a damping effect to prevent weight drift; and the third term, $-c|\hat{W}{|}^{\beta }\hat{W}$, acts as the predefined-time convergence component, guaranteeing that the weights converge within the specified time frame.*

#### 3.2.3. Predefined-Time Actual Controller

The actual control law is designed as:

(42) $$u=\frac{\alpha_{2}}{g_{1}}$$

where

(43) $$\alpha_{2}=-\frac{z_{2}{\overset{ˇ}{\alpha }}_{2}^{2}}{\sqrt{z_{2}^{2}{\overset{ˇ}{\alpha }}_{2}^{2}+\varepsilon_{2}^{2}}}$$

with $\varepsilon_{2}>0$ and

(44) $$\begin{array}{cc} \;\;\;\;\;\;\;\;\;\;\;\;\;\;\;\;\;\;{\overset{ˇ}{\alpha }}_{2}= & \frac{\pi }{\beta T_{c}}\left[2^{\beta }{\left(\frac{1}{2}\right)}^{1+\frac{\beta }{2}}{\mathrm{sig}}^{1+\beta }\left(z_{2}\right)+{\left(\frac{1}{2}\right)}^{1-\frac{\beta }{2}}{\mathrm{sig}}^{1-\beta }\left(z_{2}\right)\right] \end{array}$$

(45) $$\begin{array}{c} +{\hat{W}}_{a}^{T}S_{a}\tanh\left(\frac{z_{2}∥S_{a}∥}{\rho }\right)+K_{2}z_{2}+z_{1} \end{array}$$

where $K_{2}>0$ is a feedback gain.

**Remark** **5.** *The control law defined in Equations (42)–(45) comprises three key components: a predefined-time convergence term that ensures the tracking error converges within the specified time $T_{c}$; a neural network compensation term, ${\hat{W}}_{a}^{T}S_{a}\tanh\left(\cdot \right)$, which provides online compensation for unknown system dynamics; and stabilizing feedback terms, $K_{2}z_{2}+z_{1}$, designed to enhance closed-loop stability.*

**Remark** **6.** *The proposed Actor-Critic framework is rooted in the Adaptive Dynamic Programming (ADP) paradigm [16,17,18]. Specifically, the Critic network approximates the value function I(t) associated with the Hamilton–Jacobi–Bellman equation:*

(46) $$0=\varphi \left(t\right)+\nabla I^{T}\dot{x}$$

*where $\varphi \left(t\right)=z_{1}^{T}Dz_{1}+\tau^{T}R\tau$ is the instantaneous cost that penalizes both tracking error and control effort. The TD error $\gamma \left(t\right)=\varphi \left(t\right)+\dot{\hat{I}}\left(t\right)$ measures the discrepancy between the current value estimate and the Bellman optimality condition. Minimizing $\gamma^{2}$ drives the Critic toward the true value function.*

*The term $K_{I}\hat{I}S_{a}$ in the Actor update law (41) can be interpreted as an approximate policy gradient step: it adjusts the Actor weights in a direction that reduces the estimated long-term cost $\hat{I}$, analogous to the policy improvement step in policy iteration methods. Together with the error-driven gradient term $r_{a}z_{2}g_{1}S_{a}\tanh\left(\cdot \right)$, the Actor update simultaneously ensures Lyapunov stability (via error reduction) and approximate optimality (via cost minimization).*

*It should be noted that due to the integration with predefined-time stability requirements, the damping term $-\varsigma_{a}{\hat{W}}_{a}$ and the predefined-time term $-c_{a}{\left|{\hat{W}}_{a}\right|}^{\beta }{\hat{W}}_{a}$ modify the pure policy gradient direction. Therefore, the optimality guarantee is approximate rather than exact, representing a meaningful design trade-off between guaranteed predefined-time convergence and strict optimality. This is consistent with the ADP literature where stability-constrained policy optimization yields near-optimal rather than globally optimal policies.*

## 4. Stability Analysis

**Theorem** **1.** *Consider the single-link manipulator system (2) satisfying Assumption 1. Under the virtual controller (26), the actual controller (42), and the Actor-Critic neural network weight update laws (39) and (41), if the design parameters satisfy:*

- *0<β<1, $T_{c}>0$*
-
$\varepsilon_{1},\varepsilon_{2},\rho >0$

-
$\sigma_{c},r_{a},K_{I},K_{2}>0$

*then the closed-loop system is practically predefined-time stable (PPTS). Specifically:*

*(i)*  *The error signals $\kappa ={\left[z_{1},z_{2},{\tilde{W}}_{a},{\tilde{W}}_{c}\right]}^{T}$ converge to a compact set within the predefined time $T_{P}<T_{\max}=\sqrt{2}T_{c}$.*
*(ii)*  *All signals in the closed-loop system remain bounded.*
*(iii)*  *The convergence region is given by:*
(47) $$\Delta =\left\{\lim_{t\to T_{P}}\kappa |V\leq \min\left\{{\left(\frac{2\beta T_{c}\sigma }{\pi }\right)}^{\frac{2}{2-\beta }},{\left(\frac{2\beta T_{c}\sigma }{\pi }\right)}^{\frac{2}{2+\beta }}\right\}\right\}$$

**Proof.**  Consider the following Lyapunov function candidate:

(48) $$V=\frac{1}{2}z_{1}^{2}+\frac{1}{2}z_{2}^{2}+\frac{1}{2r_{a}}{\tilde{W}}_{a}^{T}{\tilde{W}}_{a}+\frac{1}{2r_{c}}{\tilde{W}}_{c}^{T}{\tilde{W}}_{c}$$

where ${\tilde{W}}_{a}=W_{a}^{*}-{\hat{W}}_{a}$ and ${\tilde{W}}_{c}=W_{c}^{*}-{\hat{W}}_{c}$ are the weight estimation errors.

From (31), we have:

(49) $${\dot{V}}_{1}<-\frac{\pi }{\beta T_{c}}{\left(\frac{z_{1}^{2}}{2}\right)}^{1-\frac{\beta }{2}}-\frac{\pi }{\beta T_{c}}2^{\beta }{\left(\frac{z_{1}^{2}}{2}\right)}^{1+\frac{\beta }{2}}+z_{1}z_{2}+\varepsilon_{1}$$

Taking the derivative of $V_{2}=\frac{1}{2}z_{2}^{2}$:

(50) $${\dot{V}}_{2}=z_{2}{\dot{z}}_{2}=z_{2}\left(f-{\dot{\alpha }}_{1}+g_{1}u\right)=z_{2}\left(\varphi -z_{1}-\frac{1}{2}z_{2}+g_{1}u\right)$$

Using the neural network approximation (40):

(51) $$z_{2}\varphi =z_{2}W_{a}^{*T}S_{a}+z_{2}\epsilon_{a}$$

Applying Lemma 1:

(52) $$z_{2}g_{1}W_{a}^{*T}S_{a}\leq |z_{2}|g_{1}∥W_{a}^{*}∥∥S_{a}∥\leq g_{1}∥W_{a}^{*}∥\left(\delta \rho +z_{2}∥S_{a}∥\tanh\left(\frac{z_{2}g_{1}∥S_{a}∥}{\rho }\right)\right)$$

Define $\theta_{a}=\frac{∥W_{a}^{*}∥}{g_{1}}$. Substituting the control law (42):

(53) $$z_{2}g_{1}u=z_{2}\alpha_{2}=-\frac{z_{2}^{2}{\overset{ˇ}{\alpha }}_{2}^{2}}{\sqrt{z_{2}^{2}{\overset{ˇ}{\alpha }}_{2}^{2}+\varepsilon_{2}^{2}}}$$

By Lemma 5:

(54) $$z_{2}g_{1}u<\varepsilon_{2}-\left|z_{2}{\overset{ˇ}{\alpha }}_{2}\right|$$

Expanding ${\overset{ˇ}{\alpha }}_{2}$ and combining terms:

(55) $$\begin{array}{cc} {\dot{V}}_{2} & <-\frac{\pi }{\beta T_{c}}{\left(\frac{z_{2}^{2}}{2}\right)}^{1-\frac{\beta }{2}}-\frac{\pi }{\beta T_{c}}2^{\beta }{\left(\frac{z_{2}^{2}}{2}\right)}^{1+\frac{\beta }{2}}-z_{1}z_{2}\\ \; & \;+z_{2}g_{1}{\tilde{W}}_{a}^{T}S_{a}\tanh\left(\frac{z_{2}g_{1}∥S_{a}∥}{\rho }\right)+\sigma_{2} \end{array}$$

where $\sigma_{2}=\varepsilon_{2}+\delta \theta_{a}g_{1}\rho +\frac{1}{2}{\bar{\epsilon }}_{a}^{2}$.

Taking the derivative of $V_{a}=\frac{1}{2r_{a}}{\tilde{W}}_{a}^{T}{\tilde{W}}_{a}$:

(56) $${\dot{V}}_{a}=-\frac{1}{r_{a}}{\tilde{W}}_{a}^{T}{\dot{\hat{W}}}_{a}$$

Substituting the Actor weight update law (41):

(57) $$\begin{array}{cc} {\dot{V}}_{a} & =-z_{2}g_{1}{\tilde{W}}_{a}^{T}S_{a}\tanh\left(\frac{z_{2}g_{1}∥S_{a}∥}{\rho }\right)+\frac{\varsigma_{a}}{r_{a}}{\tilde{W}}_{a}^{T}{\hat{W}}_{a}+\frac{c_{a}}{r_{a}}{\tilde{W}}_{a}^{T}{\left|{\hat{W}}_{a}\right|}^{\beta }{\hat{W}}_{a}-\frac{K_{I}}{r_{a}}{\tilde{W}}_{a}^{T}\hat{I}S_{a} \end{array}$$

Using Young’s inequality for ${\tilde{W}}_{a}^{T}{\hat{W}}_{a}={\tilde{W}}_{a}^{T}\left(W_{a}^{*}-{\tilde{W}}_{a}\right)$:

(58) $${\tilde{W}}_{a}^{T}{\hat{W}}_{a}\leq -\frac{1}{2}∥{\tilde{W}}_{a}{∥}^{2}+\frac{1}{2}{∥W_{a}^{*}∥}^{2}$$

Using Lemma 3 for ${\tilde{W}}_{a}^{T}{\left|{\hat{W}}_{a}\right|}^{\beta }{\hat{W}}_{a}$:

(59) $${\tilde{W}}_{a}^{T}|{\hat{W}}_{a}{|}^{\beta }{\hat{W}}_{a}\leq \frac{1+\beta }{2+\beta }∥W_{a}^{*}{∥}^{2+\beta }-\frac{1+\beta }{2+\beta }{∥{\tilde{W}}_{a}∥}^{2+\beta }$$

Therefore:

(60) $${\dot{V}}_{a}\leq -z_{2}g_{1}{\tilde{W}}_{a}^{T}S_{a}\tanh\left(\cdot \right)-\frac{\varsigma_{a}}{2r_{a}}∥{\tilde{W}}_{a}{∥}^{2}-\frac{c_{a}\left(1+\beta \right)}{r_{a}\left(2+\beta \right)}{∥{\tilde{W}}_{a}∥}^{2+\beta }+\sigma_{a}$$

From the definition $V_{a}=\frac{1}{2r_{a}}{∥{\tilde{W}}_{a}∥}^{2}$, we have:

(61) $$∥{\tilde{W}}_{a}{∥}^{2+\beta }={\left(2r_{a}V_{a}\right)}^{1+\frac{\beta }{2}}={\left(2r_{a}\right)}^{1+\frac{\beta }{2}}V_{a}^{1+\frac{\beta }{2}}$$

Substituting into the third term of (60):

(62) $$\begin{array}{cc} -\frac{c_{a}\left(1+\beta \right)}{r_{a}\left(2+\beta \right)}{∥{\tilde{W}}_{a}∥}^{2+\beta }\; & =-\frac{c_{a}\left(1+\beta \right)}{r_{a}\left(2+\beta \right)}{\left(2r_{a}\right)}^{1+\frac{\beta }{2}}V_{a}^{1+\frac{\beta }{2}}\\ \; & =-\frac{c_{a}\left(1+\beta \right)\cdot 2^{1+\frac{\beta }{2}}\cdot r_{a}^{\frac{\beta }{2}}}{2+\beta }V_{a}^{1+\frac{\beta }{2}} \end{array}$$

To achieve the target form $-\frac{\pi }{\beta T_{c}}2^{\frac{\beta }{2}}V_{a}^{1+\frac{\beta }{2}}$, we require:

(63) $$\frac{c_{a}\left(1+\beta \right)\cdot 2^{1+\frac{\beta }{2}}\cdot r_{a}^{\frac{\beta }{2}}}{2+\beta }=\frac{\pi }{\beta T_{c}}2^{\frac{\beta }{2}}$$

Solving for $c_{a}$:

(64) $$c_{a}=\frac{\pi (2+\beta )}{2\beta T_{c}\left(1+\beta \right)r_{a}^{\frac{\beta }{2}}}$$

With this choice of $c_{a}$, we obtain:

(65) $$-\frac{c_{a}\left(1+\beta \right)}{r_{a}\left(2+\beta \right)}{∥{\tilde{W}}_{a}∥}^{2+\beta }=-\frac{\pi }{\beta T_{c}}2^{\frac{\beta }{2}}V_{a}^{1+\frac{\beta }{2}}$$

From the definition $V_{a}=\frac{1}{2r_{a}}{∥{\tilde{W}}_{a}∥}^{2}$, we have:

(66) $$∥{\tilde{W}}_{a}{∥}^{2-\beta }={\left(2r_{a}V_{a}\right)}^{1-\frac{\beta }{2}}={\left(2r_{a}\right)}^{1-\frac{\beta }{2}}V_{a}^{1-\frac{\beta }{2}}$$

Applying Lemma 6 with $x=∥{\tilde{W}}_{a}∥$:

(67) $$∥{\tilde{W}}_{a}{∥}^{2}\geq {∥{\tilde{W}}_{a}∥}^{2-\beta }-C_{\beta }$$

Multiplying both sides by $\frac{\varsigma_{a}}{2r_{a}}$:

(68) $$\frac{\varsigma_{a}}{2r_{a}}∥{\tilde{W}}_{a}{∥}^{2}\geq \frac{\varsigma_{a}}{2r_{a}}{∥{\tilde{W}}_{a}∥}^{2-\beta }-\frac{\varsigma_{a}C_{\beta }}{2r_{a}}$$

Substituting (66):

(69) $$\begin{array}{cc} \frac{\varsigma_{a}}{2r_{a}}{∥{\tilde{W}}_{a}∥}^{2} & \geq \frac{\varsigma_{a}}{2r_{a}}{\left(2r_{a}\right)}^{1-\frac{\beta }{2}}V_{a}^{1-\frac{\beta }{2}}-\frac{\varsigma_{a}C_{\beta }}{2r_{a}}\\ \; & =\frac{\varsigma_{a}{\left(2r_{a}\right)}^{1-\frac{\beta }{2}}}{2r_{a}}V_{a}^{1-\frac{\beta }{2}}-\sigma_{a1}\\ \; & =\frac{\varsigma_{a}}{{\left(2r_{a}\right)}^{\frac{\beta }{2}}}V_{a}^{1-\frac{\beta }{2}}-\sigma_{a1} \end{array}$$

where $\sigma_{a1}=\frac{\varsigma_{a}C_{\beta }}{2r_{a}}$ is a bounded positive constant.

Therefore:

(70) $$-\frac{\varsigma_{a}}{2r_{a}}{∥{\tilde{W}}_{a}∥}^{2}\leq -\frac{\varsigma_{a}}{{\left(2r_{a}\right)}^{\frac{\beta }{2}}}V_{a}^{1-\frac{\beta }{2}}+\sigma_{a1}$$

To achieve the target form $-\frac{\pi }{\beta T_{c}}V_{a}^{1-\frac{\beta }{2}}$, we require:

(71) $$\frac{\varsigma_{a}}{{\left(2r_{a}\right)}^{\frac{\beta }{2}}}=\frac{\pi }{\beta T_{c}}$$

Solving for $\varsigma_{a}$:

(72) $$\varsigma_{a}=\frac{\pi {\left(2r_{a}\right)}^{\frac{\beta }{2}}}{\beta T_{c}}=\frac{\pi \cdot 2^{\frac{\beta }{2}}\cdot r_{a}^{\frac{\beta }{2}}}{\beta T_{c}}$$

With this choice of $\varsigma_{a}$, we obtain:

(73) $$-\frac{\varsigma_{a}}{2r_{a}}{∥{\tilde{W}}_{a}∥}^{2}\leq -\frac{\pi }{\beta T_{c}}V_{a}^{1-\frac{\beta }{2}}+\sigma_{a1}$$

Therefore, substituting (73) and (65) into (60):

(74) $${\dot{V}}_{a}\leq -z_{2}g_{1}{\tilde{W}}_{a}^{T}S_{a}\tanh\left(\cdot \right)-\frac{\pi }{\beta T_{c}}V_{a}^{1-\frac{\beta }{2}}-\frac{\pi }{\beta T_{c}}2^{\frac{\beta }{2}}V_{a}^{1+\frac{\beta }{2}}+\sigma_{a}\prime$$

where $\sigma_{a}\prime =\sigma_{a}+\sigma_{a1}$ is a bounded positive constant.

By the definitions of $\varsigma_{a}$ and $c_{a}$, and applying Lemma 6:

(75) $${\dot{V}}_{a}\leq -z_{2}g_{1}{\tilde{W}}_{a}^{T}S_{a}\tanh\left(\cdot \right)-\frac{\pi }{\beta T_{c}}{\left(\frac{∥{\tilde{W}}_{a}{∥}^{2}}{2r_{a}}\right)}^{1-\frac{\beta }{2}}-\frac{\pi }{\beta T_{c}}2^{\frac{\beta }{2}}{\left(\frac{∥{\tilde{W}}_{a}{∥}^{2}}{2r_{a}}\right)}^{1+\frac{\beta }{2}}+\sigma_{a}\prime$$

From ${\dot{V}}_{2}$, the cross term involving the Actor network is $z_{2}g_{1}{\tilde{W}}_{a}^{T}S_{a}\tanh\left(\frac{z_{2}g_{1}∥S_{a}∥}{\rho }\right)$, which arises because the control law $u=\alpha_{2}/g_{1}$ yields $z_{2}g_{1}u=z_{2}\alpha_{2}$ and the neural network compensation term in $\alpha_{2}$ contains ${\hat{W}}_{a}^{T}S_{a}\tanh\left(\cdot \right)$. From ${\dot{V}}_{a}=-\frac{1}{r_{a}}{\tilde{W}}_{a}^{T}{\dot{\hat{W}}}_{a}$, substituting the Actor update law (41), the first term is $-z_{2}g_{1}{\tilde{W}}_{a}^{T}S_{a}\tanh\left(\frac{z_{2}g_{1}∥S_{a}∥}{\rho }\right)$, these two terms cancel *exactly* for any $g_{1}>0$.

Similarly, for the Critic network:

(76) $${\dot{V}}_{c}\leq -\frac{\pi }{\beta T_{c}}{\left(\frac{∥{\tilde{W}}_{c}{∥}^{2}}{2r_{c}}\right)}^{1-\frac{\beta }{2}}-\frac{\pi }{\beta T_{c}}2^{\frac{\beta }{2}}{\left(\frac{∥{\tilde{W}}_{c}{∥}^{2}}{2r_{c}}\right)}^{1+\frac{\beta }{2}}+\sigma_{c}\prime$$

where $\sigma_{c}\prime =\sigma_{c}+\sigma_{c1}$ is a bounded positive constant.

Combining all terms:

(77) $$\dot{V}={\dot{V}}_{1}+{\dot{V}}_{2}+{\dot{V}}_{a}+{\dot{V}}_{c}$$

Note that the cross terms cancel:

- $z_{1}z_{2}$ from ${\dot{V}}_{1}$ cancels with $-z_{1}z_{2}$ from ${\dot{V}}_{2}$.
- $z_{2}g_{1}{\tilde{W}}_{a}^{T}S_{a}\tanh\left(\cdot \right)$ from ${\dot{V}}_{2}$ cancels exactly with $-z_{2}g_{1}{\tilde{W}}_{a}^{T}S_{a}\tanh\left(\cdot \right)$ from ${\dot{V}}_{a}$, since the Actor weight update law (41) explicitly includes the factor $g_{1}$ in the gradient term, and the control law $u=\alpha_{2}/g_{1}$ ensures that $z_{2}g_{1}u=z_{2}\alpha_{2}$. This exact cancellation holds for any $g_{1}=1/I>0$ without requiring any approximation.

Therefore:

(78) $$\begin{array}{cc} \dot{V} & <-\frac{\pi }{\beta T_{c}}\left[{\left(\frac{z_{1}^{2}}{2}\right)}^{1-\frac{\beta }{2}}+{\left(\frac{z_{2}^{2}}{2}\right)}^{1-\frac{\beta }{2}}+{\left(\frac{∥{\tilde{W}}_{a}{∥}^{2}}{2r_{a}}\right)}^{1-\frac{\beta }{2}}+{\left(\frac{∥{\tilde{W}}_{c}{∥}^{2}}{2r_{c}}\right)}^{1-\frac{\beta }{2}}\right]\\ \; & \;-\frac{\pi }{\beta T_{c}}2^{\frac{\beta }{2}}\left[{\left(\frac{z_{1}^{2}}{2}\right)}^{1+\frac{\beta }{2}}+{\left(\frac{z_{2}^{2}}{2}\right)}^{1+\frac{\beta }{2}}+{\left(\frac{∥{\tilde{W}}_{a}{∥}^{2}}{2r_{a}}\right)}^{1+\frac{\beta }{2}}+{\left(\frac{∥{\tilde{W}}_{c}{∥}^{2}}{2r_{c}}\right)}^{1+\frac{\beta }{2}}\right]+\sigma \end{array}$$

where $\sigma =\varepsilon_{1}+\sigma_{2}+\sigma_{a}\prime +\sigma_{c}\prime$ is a positive constant.

Applying Lemma 2, for $0<1-\frac{\beta }{2}<1$:

(79) $$\sum_{i}{\left(\frac{x_{i}^{2}}{2}\right)}^{1-\frac{\beta }{2}}\geq {\left(\sum_{i}\frac{x_{i}^{2}}{2}\right)}^{1-\frac{\beta }{2}}=V^{1-\frac{\beta }{2}}$$

for $1+\frac{\beta }{2}>1$:

(80) $$\sum_{i}{\left(\frac{x_{i}^{2}}{2}\right)}^{1+\frac{\beta }{2}}\geq 4^{-\frac{\beta }{2}}V^{1+\frac{\beta }{2}}$$

Therefore:

(81) $$\dot{V}\leq -\frac{\pi }{\beta T_{c}}\left(V^{1-\frac{\beta }{2}}+V^{1+\frac{\beta }{2}}\right)+\sigma$$

By Lemma 4, the system is practically predefined-time stable with settling time $T_{P}<T_{\max}=\sqrt{2}T_{c}$.

From the predefined-time stability, $z_{1}$, $z_{2}$, ${\tilde{W}}_{a}$, ${\tilde{W}}_{c}$ are all bounded.

This completes the proof. □

**Remark** **7.** *By adjusting the predefined time parameter $T_{c}$, the upper bound of the settling time can be explicitly preset: $T_{P}<T_{\max}=\sqrt{2}T_{c}$. A smaller $T_{c}$ leads to faster convergence but may require larger control efforts.*

**Remark** **8.** *The predefined-time parameters for both Actor and Critic networks are derived from the requirement that the weight estimation error dynamics satisfy the predefined-time stability condition in Lemma 4. The key insight is:*

- *The damping term $-\varsigma \hat{W}$ generates the $V^{1-\frac{\beta }{2}}$ component through Lemma 6, which dominates when V is large.*
- *The predefined-time term $-c|\hat{W}{|}^{\beta }\hat{W}$ directly generates the $V^{1+\frac{\beta }{2}}$ component through algebraic substitution, which dominates when V is small.*
- *The combination of both terms ensures predefined-time convergence for all values of V>0.*

## 5. Simulation Results

In this section, numerical simulations are conducted to verify the effectiveness of the proposed Actor-Critic predefined-time control scheme. The simulations are performed on a single-link manipulator system using MATLAB R2025a with Runge–Kutta 4th order integration.

### 5.1. Simulation Setup

The initial conditions are set as $x_{1}\left(0\right)=1.0$ rad and $x_{2}\left(0\right)=-1.0$ rad/s. The simulation runs for 20 s with a step size of Δt=0.001 s. For the Actor network with 100 nodes ($l_{a}=100$) processing 5-dimensional input $\left[x_{1},x_{2},y_{d},{\dot{y}}_{d},{\ddot{y}}_{d}\right]$, the basis centers are uniformly sampled from the hypercube ${\left[-3,3\right]}^{5}$ with width parameter $\eta_{a}=1.2$. All weights are initialized to zero, ${\hat{W}}_{a}\left(0\right)=0\in R^{100}$, and bounded by $|{\hat{W}}_{a,i}|\leq 200$ via saturation clipping. The Critic network uses 64 nodes ($l_{c}=64$) with 2-dimensional input $\left[z_{1},z_{2}\right]$. Centers are placed on an 8×8 uniform grid over ${\left[-2,2\right]}^{2}$ with width $\eta_{c}=1.0$. Weights are similarly initialized as ${\hat{W}}_{c}\left(0\right)=0\in R^{64}$ and bounded by $|{\hat{W}}_{c,i}|\leq 100$. Regarding the discount factor, we implement ψ=10 rather than the theoretical limit ψ→∞. This is a standard simplification in ADP literature [18], where using a sufficiently large ψ makes the term $-S_{c}/\psi$ negligible compared to $\nabla S_{c}\cdot Z_{c}$, effectively approximating the infinite discount case while maintaining numerical stability. The system and control parameters are given in Table 1.

To verify that the performance is not an artifact of a specific initial condition, we additionally conducted 20 independent Monte Carlo simulations with randomized initial conditions uniformly drawn from $x_{1}\left(0\right)\in \left[0.5,1.5\right]$ rad and $x_{2}\left(0\right)\in \left[-1.5,-0.5\right]$ rad/s. The statistical results are reported in Table 2.

### 5.2. Tracking Performance Analysis

**Figure 2:**
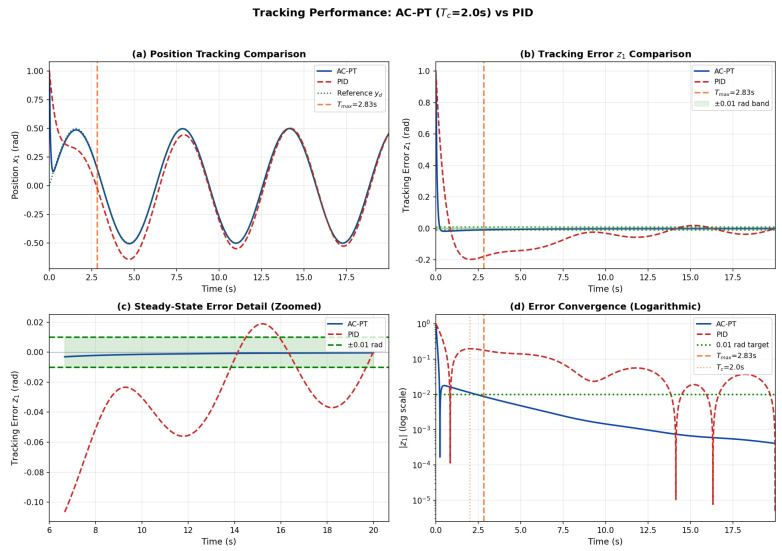
Tracking performance comparison: (**a**) Position tracking showing both controllers following the reference trajectory; (**b**) Tracking error $z_{1}$ with ±0.01 rad tolerance band; (**c**) Steady-state error detail (zoomed view after t>6 s); (**d**) Error convergence in logarithmic scale showing the convergence rate.

Figure 2 compares the tracking performance of the proposed AC-PT controller and the conventional PID controller. As shown in Figure 2a, both controllers track the reference trajectory $y_{d}=0.5\sin\left(t\right)$, but the AC-PT controller achieves stabilization within approximately 0.23 s, well within the theoretical upper bound $T_{\max}=2.83$ s. In contrast, the PID controller requires approximately 13.84 s to reach the ±0.01 rad tolerance band (Figure 2b). The zoomed steady-state view in Figure 2c confirms that the AC-PT controller maintains the tracking error consistently within the specified tolerance, whereas the PID controller exhibits noticeable residual oscillations. The logarithmic-scale convergence plot in Figure 2d further illustrates the characteristic rapid error decay before $T_{\max}$, corroborating the predefined-time stability guarantee of Theorem 1. The quantitative comparison is summarized in Table 3: the AC-PT controller achieves 96.9% reduction in steady-state RMSE and 98.3% reduction in settling time compared to PID control.

### 5.3. Neural Network Learning Process

**Figure 3:**
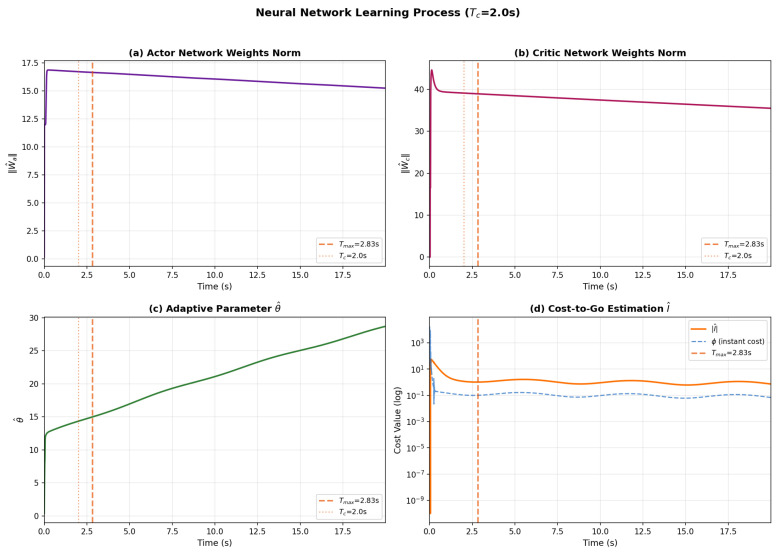
Neural network learning process: (**a**) Actor network weight norm $∥{\hat{W}}_{a}∥$; (**b**) Critic network weight norm $∥{\hat{W}}_{c}∥$; (**c**) Adaptive parameter $\hat{\theta }$; (**d**) Cost-to-go estimation $\hat{I}$ and instantaneous cost ϕ.

The online learning behavior of the Actor-Critic neural networks is shown in Figure 3. Both the Actor and Critic weight norms (Figure 3a,b) converge to bounded values and remain stable throughout the simulation, confirming that the predefined-time weight update laws incorporating the $|\hat{W}{|}^{\beta }\hat{W}$ terms successfully prevent weight divergence. The adaptive parameter $\hat{\theta }$ (Figure 3c) increases during the transient phase to compensate for system uncertainties and subsequently stabilizes as the tracking error diminishes. Figure 3d shows that both the cost-to-go estimation $\hat{I}$ and the instantaneous cost ϕ decrease rapidly during the initial phase, indicating that the Actor-Critic framework effectively optimizes the control policy while compensating for unknown system dynamics.

### 5.4. Effect of Predefined Time Parameter

**Figure 4:**
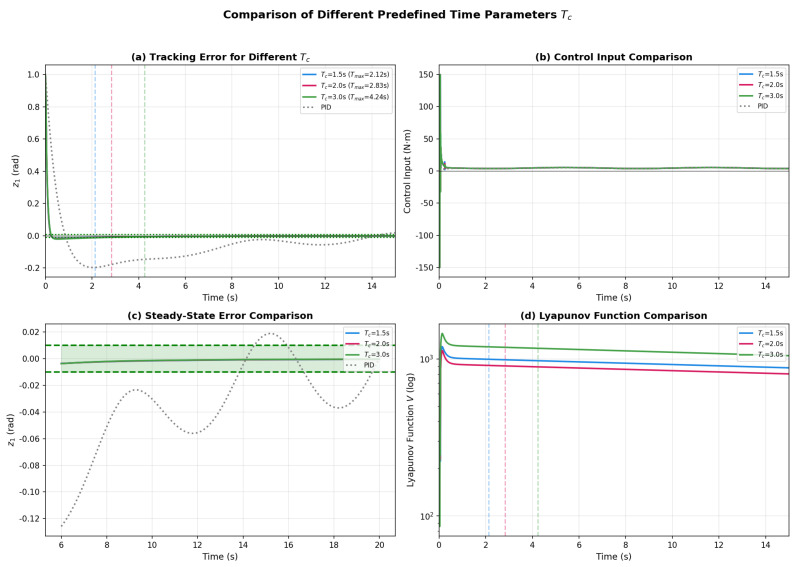
Comparison of different predefined time parameters: (**a**) Tracking error for different $T_{c}$; (**b**) Control input comparison; (**c**) Steady-state error comparison; (**d**) Lyapunov function evolution.

The influence of the predefined time parameter $T_{c}$ on control performance is investigated through simulations with $T_{c}\in \left\{1.5,2.0,3.0\right\}$ s, as shown in Figure 4. Smaller $T_{c}$ values lead to faster error convergence (Figure 4a), with the system converging before $T_{\max}=2.12$ s for $T_{c}=1.5$ s. However, this faster convergence comes at the cost of larger initial control effort (Figure 4b), presenting a trade-off between convergence speed and actuator requirements. Figure 4c demonstrates that all tested $T_{c}$ values achieve comparable steady-state accuracy, indicating that $T_{c}$ primarily governs the transient response rather than the ultimate tracking precision. The Lyapunov function evolution in Figure 4d confirms that V(t) decreases below its corresponding $T_{\max}$ bound in all cases, thereby validating the predefined-time stability theory of Theorem 1 across different parameter settings.

### 5.5. Comparison with State-of-the-Art Methods

**Figure 5:**
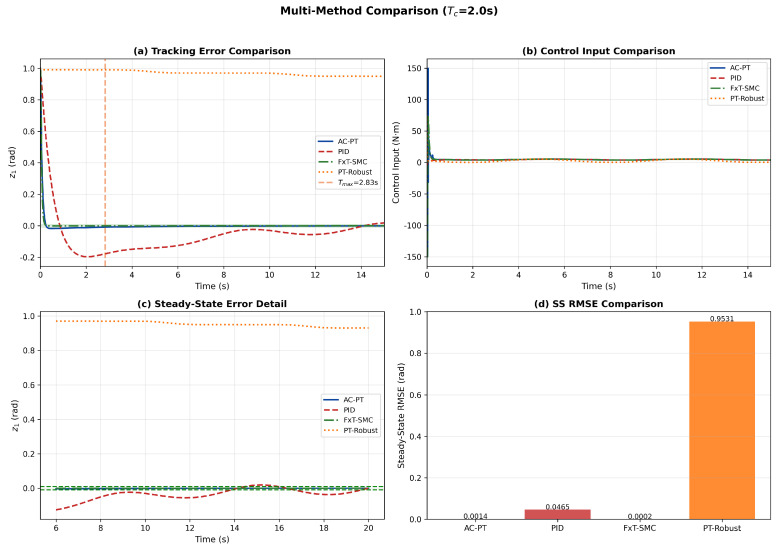
Comparison of multiple methods: (**a**) Tracking error comparison; (**b**) Control input comparison; (**c**) Steady-state error comparison; (**d**) SS RMSE Comparison.

To further substantiate the contributions, the proposed AC-PT controller is compared with two representative methods from the literature: a disturbance-observer-based fixed-time sliding mode controller (FxT-SMC) based on [26], and a predefined-time robust controller without neural networks (PT-Robust) based on [24]. The tracking error comparison in Figure 5a shows that all three advanced controllers significantly outperform PID, with AC-PT and PT-Robust achieving comparable transient performance. The steady-state error detail in Figure 5c reveals that AC-PT achieves the smallest residual error among all methods. The quantitative results demonstrate that the proposed AC-PT method provides competitive convergence speed while offering two key advantages: online learning capability for unknown dynamics compensation (absent in PT-Robust) and an explicit, user-tunable settling time bound $T_{P}<\sqrt{2}\;T_{c}$ (which FxT-SMC cannot directly prescribe).

### 5.6. Robustness Evaluation

To evaluate the robustness of the proposed AC-PT controller under model uncertainties, we conduct simulations under three categories of perturbations: (i) mass uncertainty (*m* varied by ±30%), (ii) friction coefficient uncertainty ($c_{0}$ varied by ±50%), and (iii) increased external disturbance (amplitude scaled to 2×, 3×, and 5× nominal). All tests use the baseline initial condition $x_{1}\left(0\right)=1.0$ rad, $x_{2}\left(0\right)=-1.0$ rad/s with all controller parameters unchanged from Table 1.

The results are summarized in Table 4. The AC-PT controller satisfies the predefined-time guarantee $T_{P}<T_{\max}=2.83$ s in all tested scenarios without any parameter re-tuning. The settling time remains within the narrow range [0.207,0.212] s, and the steady-state RMSE is maintained at approximately 0.0015 rad across all cases.

**Figure 6:**
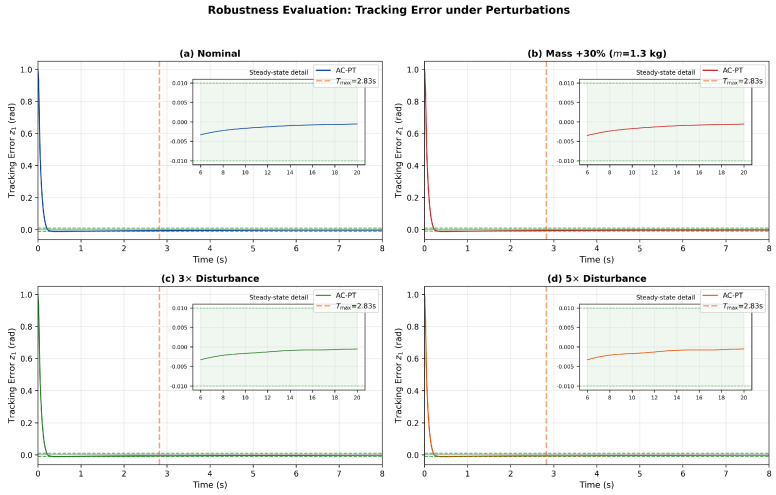
Robustness evaluation: tracking error $z_{1}$ under parameter perturbations. (**a**) Nominal parameters; (**b**) Mass increased by 30% (m=1.3 kg); (**c**) Disturbance amplitude tripled (3×d(t)); (**d**) Disturbance amplitude quintupled (5×d(t)). The dashed vertical line indicates $T_{\max}=2.83$ s. The green band denotes the ±0.01 rad tolerance. All scenarios satisfy $T_{P}<T_{\max}$.

This strong invariance is theoretically grounded: the predefined-time convergence rate in Lemma 4 depends on the control gains $K_{1,\mathrm{pt}}$, $K_{2,\mathrm{pt}}$ and the design parameter $T_{c}$, which are independent of the physical parameters. The adaptive parameter $\hat{\theta }$ and the Actor neural network compensate for the parametric variations and disturbance changes online, as predicted by Theorem 1. The representative tracking error trajectories in Figure 6 confirm that the convergence behavior is qualitatively preserved under all perturbation conditions.

## 6. Conclusions

This paper has presented a predefined-time adaptive neural tracking control framework for uncertain single-link manipulator systems, integrating predefined-time stability theory with an Actor-Critic reinforcement learning architecture. The main contribution lies in the synergistic design where the predefined-time convergence mechanism is incorporated into both the control law and the neural network weight update laws, enabling a single parameter $T_{c}$ to explicitly prescribe the settling time upper bound as $T_{P}<\sqrt{2}T_{c}$, independent of initial conditions and system parameters.

The current work has several limitations that motivate future research. First, the single-link manipulator setting does not capture the coupling effects present in multi-DOF systems; extending the framework to multi-link and redundant manipulators with inter-joint coupling is a natural next step. Second, the current validation is simulation-based; experimental validation on physical robot platforms is essential to assess real-world applicability. Additional future directions include: incorporating input saturation constraints and actuator dynamics; developing event-triggered implementations to reduce computational and communication overhead.

## Figures and Tables

**Table 1:** System and Control Parameters.

Parameter	Description	Value	Unit
System Parameters			
*m*	Link mass	1.0	kg
*l*	Link length	0.5	m
$c_{0}$	Friction coefficient	1.0	N·m·s/rad
*g*	Gravitational acceleration	9.8	m/s^2^
d(t)	External disturbance	0.5sin(t)+0.3cos(2t)	N·m
$y_{d}\left(t\right)$	Reference trajectory	0.5sin(t)	rad
Predefined-Time Parameters			
$T_{c}$	Predefined time parameter	2.0	s
$T_{\max}$	Maximum settling time	$\sqrt{2}T_{c}=2.83$	s
β	Convergence parameter	0.6	-
Controller Parameters			
$K_{2}$	Feedback gain	100	-
$\varepsilon_{1},\varepsilon_{2}$	Small constants	$10^{-4}$	-
ρ	Smoothing parameter	0.05	-
Neural Network Parameters			
$l_{a}$	Actor network nodes	100	-
$l_{c}$	Critic network nodes	64	-
$\sigma_{a},\sigma_{c}$	Learning rates	100, 50	-
$K_{I}$	Critic feedback gain	2.0	-
$\eta_{a}$, $\eta_{c}$	RBF widths	1.2, 1.0	-
ψ	Discount factor	10	-
$W_{a}^{\max}$, $W_{c}^{\max}$	Weight bounds	200, 100	-
PID Controller			
$K_{p},K_{d},K_{i}$	PID gains	25, 12, 5	-

**Table 2:** Statistical Performance over 20 Monte Carlo Runs (Mean ± Std).

Performance Metric	AC-PT	PID	Improvement
Total RMSE (rad)	0.0417±0.0129	0.1266±0.0275	67.0%
SS RMSE (rad)	0.0014±0.0004	0.0454±0.0042	96.9%
Max SS Error (rad)	0.0030±0.0009	0.1229±0.0108	97.5%
Settling Time (s)	0.2031±0.0068	13.824±0.063	98.5%
$T_{P}<T_{\max}$ Satisfied	20/20 (100%)	N/A	—

N/A: Since the PID controller’s response is slow and does not meet the basic premise for evaluating this time constraint, this metric is not applicable.

**Table 3:** Performance Comparison: AC-PT vs PID Control ($T_{c}=2.0$ s). Single-run results with baseline initial condition $x_{1}\left(0\right)=1.0$ rad, $x_{2}\left(0\right)=-1.0$ rad/s.

Performance Metric	AC-PT	PID	Improvement
Total RMSE (rad)	0.0467	0.1333	65.0%
Steady-State RMSE (rad)	0.0014	0.0465	96.9%
Max Steady-State Error (rad)	0.0037	0.1259	97.1%
Settling Time to ±0.01 rad (s)	0.229	13.841	98.3%
Time Within ±0.01 rad (%)	100.0	12.2	-

**Table 4:** Robustness Evaluation under Parameter Perturbations ($T_{c}=2.0$ s, $T_{\max}=2.83$ s).

Scenario	Total RMSE	SS RMSE	Settling Time	$T_{P}<T_{\max}$
	**(rad)**	**(rad)**	**(s)**	**Satisfied**
Nominal (m=1.0, $c_{0}=1.0$)	0.0450	0.0015	0.209	Yes
*Mass Uncertainty*				
m=0.7 kg (−30%)	0.0429	0.0015	0.207	Yes
m=1.3 kg (+30%)	0.0469	0.0016	0.212	Yes
*Friction Uncertainty*				
$c_{0}=0.5$ (−50%)	0.0448	0.0015	0.208	Yes
$c_{0}=1.5$ (+50%)	0.0453	0.0015	0.210	Yes
*Increased Disturbance*				
2× disturbance	0.0450	0.0015	0.209	Yes
3× disturbance	0.0450	0.0015	0.209	Yes
5× disturbance	0.0450	0.0015	0.209	Yes

## Data Availability

The data presented in this study are available from the corresponding author upon reasonable request.

## References

[1] Gao H., Yang Y., Liu J., Sun C. (2025). Reinforcement Learning-Based Admittance Control for Physical Human–Robot Interaction with Output Constraints. IEEE Trans. Autom. Sci. Eng..

[2] Vyas Y.J., van der Wijk V., Cocuzza S. (2025). A Review of Mechanical Design Approaches for Balanced Robotic Manipulation. Robotics.

[3] Zhang D., Hu J., Cheng J., Wu Z.G., Yan H. (2024). A Novel Disturbance Observer Based Fixed-Time Sliding Mode Control for Robotic Manipulators with Global Fast Convergence. IEEE/CAA J. Autom. Sin..

[4] Sun Y., Yan B., Shi P., Lim C.C. (2024). Consensus for Multiagent Systems Under Output Constraints and Unknown Control Directions. IEEE Syst. J..

[5] Liu J., Wang Q.G., Yu J. (2024). Event-Triggered Adaptive Neural Network Tracking Control for Uncertain Systems with Unknown Input Saturation Based on Command Filters. IEEE Trans. Neural Netw. Learn. Syst..

[6] Li W., Zhang Z., Ge S.S. (2023). Dynamic Gain Reduced-Order Observer-Based Global Adaptive Neural-Network Tracking Control for Nonlinear Time-Delay Systems. IEEE Trans. Cybern..

[7] Xie X., Chen W., Xia C., Xing J., Chang L. (2026). An RBFNN-Based Prescribed Performance Controller for Spacecraft Proximity Operations with Collision Avoidance. Sensors.

[8] Zhang X., Li H., Zhu G., Zhang Y., Wang C., Wang Y., Su C.Y. (2024). Finite-Time Adaptive Quantized Control for Quadrotor Aerial Vehicle with Full States Constraints and Validation on QDrone Experimental Platform. Drones.

[9] Zhang S., Yang P., Kong L., Li G., He W. (2020). A Single Parameter-Based Adaptive Approach to Robotic Manipulators with Finite Time Convergence and Actuator Fault. IEEE Access.

[10] Li G., Chen X., Yu J., Liu J. (2022). Adaptive Neural Network-Based Finite-Time Impedance Control of Constrained Robotic Manipulators with Disturbance Observer. IEEE Trans. Circuits Syst. II Express Briefs.

[11] Jiménez-Rodríguez E., Muñoz-Vázquez A.J., Sánchez-Torres J.D., Defoort M., Loukianov A.G. (2020). A Lyapunov-Like Characterization of Predefined-Time Stability. IEEE Trans. Autom. Control.

[12] Zhang T., Bai R., Li Y. (2023). Practically Predefined-Time Adaptive Fuzzy Quantized Control for Nonlinear Stochastic Systems with Actuator Dead Zone. IEEE Trans. Fuzzy Syst..

[13] Liu B., Wang W., Li Y., Yi Y., Xie G. (2022). Adaptive Quantized Predefined-Time Backstepping Control for Nonlinear Strict-Feedback Systems. IEEE Trans. Circuits Syst. II Express Briefs.

[14] Xie S., Chen Q. (2022). Adaptive Nonsingular Predefined-Time Control for Attitude Stabilization of Rigid Spacecrafts. IEEE Trans. Circuits Syst. II Express Briefs.

[15] Fan Y., Yang C., Zhan H., Li Y. (2024). Neuro-Adaptive-Based Predefined-Time Smooth Control for Manipulators with Disturbance. IEEE Trans. Syst. Man Cybern. Syst..

[16] Lewis F.L., Vrabie D., Vamvoudakis K.G. (2012). Reinforcement Learning and Feedback Control: Using Natural Decision Methods to Design Optimal Adaptive Controllers. IEEE Control Syst. Mag..

[17] Ouyang Y., He W., Li X. (2017). Reinforcement learning control of a single-link flexible robotic manipulator. IET Control Theory Appl..

[18] Vamvoudakis K.G., Lewis F.L. (2010). Online actor–critic algorithm to solve the continuous-time infinite horizon optimal control problem. Automatica.

[19] Guan X., Li Y.X., Hou Z., Ahn C.K. (2025). Reinforcement Learning-Based Event-Triggered Adaptive Fixed-Time Optimal Formation Control of Multiple QAAVs. IEEE Trans. Aerosp. Electron. Syst..

[20] Liu Y.J., Li S., Tong S., Chen C.L.P. (2019). Adaptive Reinforcement Learning Control Based on Neural Approximation for Nonlinear Discrete-Time Systems with Unknown Nonaffine Dead-Zone Input. IEEE Trans. Neural Netw. Learn. Syst..

[21] Zhang Y., Liang X., Li D., Ge S.S., Gao B., Chen H., Lee T.H. (2024). Reinforcement Learning-Based Time-Synchronized Optimized Control for Affine Systems. IEEE Trans. Artif. Intell..

[22] Sun Y., Shi P., Lim C.C. (2021). Event-triggered adaptive leaderless consensus control for nonlinear multi-agent systems with unknown backlash-like hysteresis. Int. J. Robust Nonlinear Control.

[23] Hu G., Xu D., Hua W., Jiang B., Shi P., Rudas I.J. (2025). Fixed-Time Cooperative Sliding Mode Control for Synchronization of Multilinear Motor Systems. IEEE/ASME Trans. Mechatronics.

[24] Muñoz-Vázquez A.J., Sánchez-Torres J.D., Jiménez-Rodríguez E., Loukianov A.G. (2019). Predefined-time robust stabilization of robotic manipulators. IEEE/ASME Trans. Mechatronics.

[25] Sun Y., Shi P., Lim C.C. (2022). Adaptive consensus control for output-constrained nonlinear multi-agent systems with actuator faults. J. Frankl. Inst..

[26] Zhang L., Su Y., Wang Z., Wang H. (2024). Fixed-time terminal sliding mode control for uncertain robot manipulators. ISA Trans..

